# Cinnarizine, a Calcium Channel Blocker, Partially Prevents the Striatal Dopamine Decline and Loss of Nigral Dopamine Neurons in the Lactacystin-Induced Rat Model of Parkinson’s Disease

**DOI:** 10.3390/ijms26188833

**Published:** 2025-09-10

**Authors:** Elżbieta Lorenc-Koci, Tomasz Lenda, Jolanta Konieczny, Danuta Jantas, Helena Domin

**Affiliations:** Maj Institute of Pharmacology, Polish Academy of Sciences, 12 Smętna Street, 31-343 Kraków, Poland; lenda@if-pan.krakow.pl (T.L.); koniecz@if-pan.krakow.pl (J.K.); jantas@if-pan.krakow.pl (D.J.); domin@if-pan.krakow.pl (H.D.)

**Keywords:** lactacystin-induced model of Parkinson’s disease, cinnarizine, voltage-gated calcium channel blocker, dopamine metabolism, proteasome inhibitor lactacystin, substantia nigra (SN), tyrosine hydroxylase (TH)

## Abstract

Selective proteasome inhibitors, used to model Parkinsonian-like pathology, are known to disrupt calcium homeostasis, but the role of calcium ions in dopaminergic neuron degeneration remains unclear. The present in vivo study examined the effects of a 7-day intraperitoneal administration of cinnarizine (10 or 30 mg/kg), a voltage-gated calcium channel blocker, in rats unilaterally injected into the substantia nigra compacta (SNc) with lactacystin (Lac; 1 µg/2 µL) or vehicle. Dopamine (DA) and its metabolites were quantified in striatal homogenates via high-performance liquid chromatography. The SN of rats treated with 10 mg/kg cinnarizine was used for Western blot analysis of tyrosine hydroxylase (TH), while tissue from animals receiving 30 mg/kg was processed for histological analysis of TH-immunoreactive (TH-ir) and cresyl violet (CV)-stained neurons. Significant reductions in striatal DA and its metabolites were observed one week after Lac injection, along with increased DA catabolism. Cinnarizine at both doses partially prevented DA loss and attenuated enhanced DA turnover. Moreover, 10 mg/kg cinnarizine partially preserved TH protein levels, while 30 mg/kg provided histological protection of TH-ir neurons in the SN. Cinnarizine was also tested in vitro in human SH-SY5Y neuroblastoma cells and primary mouse cortical neurons exposed to Lac or rotenone to further assess its neuroprotective potential. In SH-SY5Y cells, cinnarizine (1–10 µM) significantly increased cell viability and reduced lactate dehydrogenase release after toxin exposure. Cinnarizine failed to counteract lactacystin-induced toxicity in primary cortical neurons but markedly reduced rotenone-evoked cell death at similar concentrations. These findings indicate that cinnarizine exerts dose-dependent neuroprotective effects in vivo and selective protective actions in vitro, supporting the potential utility of voltage-gated calcium channel blockers in treating Parkinson’s disease.

## 1. Introduction

The key pathological hallmark of Parkinson’s disease (PD) is the progressive degeneration of dopamine-containing (DA) neurons in the substantia nigra pars compacta (SNc), resulting in a dramatic loss of striatal DA and the formation of proteinaceous inclusions predominantly containing aggregated α-synuclein known as Lewy bodies [1]. Although the mechanisms underlying the degenerative process of DA neurons in the SN are still unclear, oxidative stress, protein mishandling, disruption of intracellular calcium (Ca^2+^) homeostasis, and mitochondrial dysfunction are mentioned as important potential pathological factors [2,3,4,5,6].

The ubiquitin-proteasome system (UPS) is the leading player in the degradation of proteins, which are involved in many biological processes [7,8], including those contributing to synapse formation [9]. Dysfunction of the UPS system, leading to the accumulation of misfolded and unwanted proteins (e.g., α-synuclein), has been reported in the SN of PD patients [10,11,12,13,14] and was proposed to be a potential etiopathogenic factor for this disease [2,11,15,16]. Therefore, in preclinical studies, in addition to the well-known neurotoxins such as 6-hydroxydopamine (6-OHDA) and 1-methyl-4-phenyl-1,2,3,6-tetrahydropyridine (MPTP), specific, selective proteasome inhibitors, mainly administered directly into the SN, were also used to reproduce biochemical and behavioral changes typical of PD in laboratory animals [17,18,19,20].

In turn, other studies suggest that disorders of Ca^2+^ homeostasis in the brain are associated with developing PD pathology [21,22,23,24]. In general, it is commonly known that Ca^2+^ is a universal and versatile second messenger that mediates numerous neuronal functions, such as the regulation of neurotransmitter release [25], neuronal excitability [26], synaptic plasticity [27], metabolism [28], gene expression [29,30,31], and apoptosis or necrosis [32,33,34]. The entry of Ca^2+^ ions into neurons and the appropriate regulation of their cytosolic concentration are essential for maintaining optimal conditions during physiological processes [27]. The neuronal Ca^2+^ homeostasis system involves various transmembrane channels [35,36], subcellular organelles [37,38,39,40], and Ca^2+^ buffering proteins [41], which act as sensors.

The influx of Ca^2+^ ions into neurons from the extracellular space is mediated by a variety of voltage-gated Ca^2+^ channels (VGCCs) and receptor-operated Ca^2+^ channels [36,38,42]. VGCCs can be classified into high- and low-voltage-activated channels belonging to three subfamilies: Cav1, Cav2, and Cav3. The Cav1 and Cav2 channel subfamilies belong to the high-voltage-activated channel group, while the Cav3 subfamily belongs to the low-voltage-activated channel group. Ca^2+^ channels of the Cav1 subfamily, also known as L-type voltage-gated Ca^2+^ channels (L-VGCCs), are widely distributed in the brains of humans and rodents [43,44,45]. Regarding the Cav1 subfamily, in the mature control brain, the Cav1.2 subtype accounts for 70%, while the Cav1.3 subtype accounts for 10–22% of its total pool [44,46,47]. However, in post-mortem studies performed on early PD brains, the ratio of Cav1.2 to Cav1.3 channels was found to differ throughout the brain compared to controls, with an increased use of Cav1.3 channels in some brain regions [44,48]. In the SNc of control subjects, Cav1.2 is the dominant subtype that colocalizes with less abundant Cav1.3 channels. In turn, in the SNc of PD patients, the number of cells that express Cav1.2 and Cav1.3 subtype channels was markedly reduced. However, the staining intensity of Cav1.3 subtype channels, assessed by optical density, increased, whereas the staining intensity of Cav1.2 remained unchanged, despite the reduced number of cells expressing both channel subtypes [44,48]. Therefore, a shift towards greater expression and utilization of Cav1.3 channels in the SNc may lead to increased Ca^2+^ influx into DA neurons and, over time, to increased mortality.

One of the characteristic features of DA neurons in the SN that degenerate in PD is their autonomous activity, resulting from prominent transmembrane Ca^2+^ currents that generate regular, slow action potentials in the absence of synaptic input [49]. The rhythmic pacemaker activity of those DA neurons maintains basal DA levels in the striatum, the main target brain region innervated by the nigral DA neurons. For a long time, the Cav1.3 subtype channels have been considered the primary driver of this pacemaker activity in the nigral DA neurons [50,51]. Furthermore, it has been strongly suggested that the age-related reliance on Cav1.3 channels to drive pacemaker-mediated increases in cytosolic Ca^2+^ concentration in the nigral DA neurons may be a dominant pathogenic factor initiating the degeneration process in PD [50]. Therefore, in preclinical studies, to protect DA neurons in the SN against the influx of excessive amounts of Ca^2+^ ions into their cytosol, the use of L-VGCC antagonists (especially those affecting Cav1.3 channels) has been proposed [22,23,50,52,53,54]. Parallel to these research objectives, the results of retrospective epidemiological studies of patients using L-VGCC antagonists (mainly dihydropyridines) in the treatment of hypertension have shown a reduced risk of developing PD [55,56,57,58,59,60]. The latter data seemed to provide a rational premise for using these drugs in the early phase of PD to limit or at least slow down its progression.

On the other hand, recently it has been shown that pacemaker activity of DA neurons in the SN is intrinsically generated not only by L-VGCC but rather by concerted interplay of a range of voltage-gated ion channels, including T-type voltage-gated Ca^2+^ channels (T-VGCC) belonging to the Cav3 subfamily [36,51]. T-VGCCs are widespread in DA neurons of the SN [61,62,63,64]. The SNc can be divided into two anatomical regions, the dorsal and ventral tiers [65], which differ in their Ca^2+^ binding protein calbindin-D28K content. The dorsal tier of SNc contains dopamine neurons with a high level of calbindin (calbindin-positive neurons; Calb^+^), which tend to be resilient to degeneration [41,66]. In contrast, the ventral tier of SNc, which lies directly above the substantia nigra reticulata, contains mainly calbindin-negative (Calb^−^) dopamine neurons devoid of this protein [41,66,67], which are susceptible to degeneration in PD. A comparison of these two parts of SNc suggests that Calb plays a significant role in protecting nigral DA neurons from death induced by excessive cytosolic Ca^2+^ concentration [68]. Interestingly, T-VGCCs were predominantly located on (Calb^−^) dopamine neurons [69]. Furthermore, T-VGCCs are associated physically and functionally with voltage- and Ca^2+^-activated potassium channels [70,71,72,73]. These associations provide Ca^2+^-dependent control of potassium channel activity via the T-type channels, which regulate neuronal firing patterns [74]. Hence, despite the abundant experimental evidence indicating the ability of L-VGCC antagonists to protect SNc DA neurons from degeneration [22,23,52,53,54], recently, the possibility of using T-VGCC antagonists in the therapy of PD as neuroprotective drugs has been widely discussed [75,76,77].

Consistent with the above-presented trend of searching for new neuroprotective therapies for PD, the present study aimed to examine whether the intraperitoneal (i.p.) administration of the dual T- and L-type Ca^2+^ channel blocker cinnarizine (Cin), with preferential activity on T-type channels [78], to rats unilaterally injected with a single dose of the selective proteasome inhibitor lactacystin (Lac) into the SNc could prevent both the degeneration of DA neuronal bodies in the SN and the loss of striatal dopamine. Subchronic treatment with two chosen doses of Cin was initiated 4 h after a unilateral intranigral injection of Lac and continued once daily for seven consecutive days. In addition, the neuroprotective properties of Cin were tested in two types of cell cultures: human neuroblastoma SH-SY5Y cells and mouse primary cortical cells, exposed to two types of cell damaging factors: Lac or rotenone (Rot). We hope this set of experimental studies will shed new light on the neuroprotective properties of VGCC antagonists in treating Parkinson’s disease.

## 2. Results

### 2.1. Effects of the In Vivo Study

#### 2.1.1. Impact of 10 and 30 mg/kg of Cinnarizine on DA and 5-HT Metabolism in the Striatum of a Lactacystin-Induced Rat Model of Parkinson’s Disease

One week after unilateral administration of Lac at a dose of 1 μg/2 μL directly into the left SNc, a significant decrease in the levels of DA and its metabolites: 3,4-dihydroxyphenylacetic acid (DOPAC), 3-methoxytyramine (3-MT), and homovanilic acid (HVA) were observed in the lesioned striatum compared to their concentration in the striatum of the control groups of rats receiving the vehicle (veh) unilaterally into the SNc and i.p. the veh or Cin (veh + veh, veh + Cin10, veh + Cin 30) at the tested doses.

**Figure 1:**
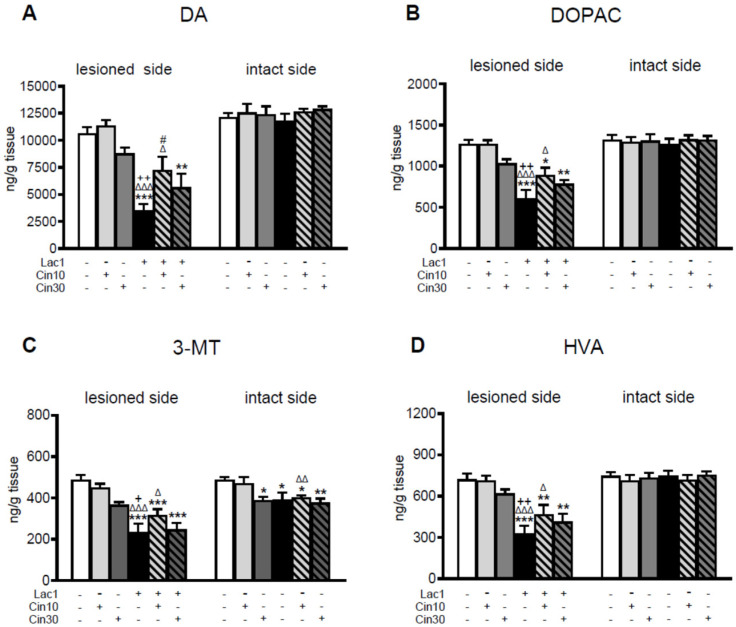
The concentration of dopamine (DA) and its metabolites (DOPAC, 3-MT, HVA) in the striatum of rats injected unilaterally into the left substantia nigra pars compacta (SNc) with a single dose of proteasome inhibitor lactacystin (Lac1; 1 μg/2 μL) or vehicle (veh) and treated i.p. with cinnarizine (Cin; 10 or 30 mg/kg) or vehicle (veh) for the first time 4 h after surgery and then once daily for seven successive days. Determination of DA and its metabolites was performed separately for the lesioned and intact sides, two hours after the last tested doses of Cin or vehicle. The data are presented as the mean ± S.E.M.; the number of rats per each experimental group was as follows: veh + veh = 10, veh + Cin10 = 8, veh + Cin30 = 6, Lac1 + veh = 10, Lac1 + Cin10 = 10, and Lac1 + Cin30 = 9. Statistical analysis of data presented in (**A**–**D**) was completed using a one-way ANOVA. Its effects in the lesioned striatum were as follows: (**A**) for DA F_(5,47)_ = 11.219, *p* < 0.0001; (**B**) for DOPAC F_(5,47_) = 11.106, *p* < 0.0001; (**C**) for 3-MT F_(5,47)_ = 13.778, *p* < 0.0001; (**D**) for HVA F_(5,47)_ = 9.765, *p* < 0.0001. Effects of one-way ANOVA in the intact striatum for DA, DOPAC, and HVA were non-significant; only for 3-MT was its effect significant, F_(5,47)_ = 4.355, *p* < 0.01. Symbols indicate the significance of differences between the tested groups according to the Newman–Keuls post hoc test: * *p* < 0.05, ** *p* < 0.01, *** *p* < 0.001 vs. veh + veh-; ^Δ^ *p* < 0.05, ^ΔΔ^ *p* < 0.01, ^ΔΔΔ^ *p* < 0.001 vs. veh + Cin10-; ^+^ *p* < 0.05, ^++^ *p* < 0.01 vs. veh + Cin30-; ^#^ *p* < 0.05 vs. Lac1 + veh-treated groups.

Such changes in the concentrations of DA, DOPAC, and HVA were not found in the intact striatum, except for the 3-MT levels, which were significantly decreased in groups veh + Cin30, Lac1 + veh, Lac1 + Cin10, and Lac1 + Cin30 compared to the veh + veh-treated group. Moreover, the concentration of 3-MT in the intact striatum of the Lac1 + Cin10-treated group was decreased compared to the corresponding group treated with veh + Cin10 on this side of the brain (Figure 1C). Cin at both examined doses partially prevented the Lac1-induced loss of tissue DA concentration in the lesioned striatum. Its concentration in this structure in rats receiving 10 mg/kg of Cin was significantly increased compared to the Lac1-treated group administered i.p. vehicle. However, it was still lower than in the control veh + veh-treated group. Administration of 30 mg/kg of Cin to the Lac1-pretreated group produced only an increasing trend in the tissue content of DA in the lesioned striatum (Figure 1A). Parallel to the increase in DA level, 10 mg/kg of Cin also enhanced the concentrations of its metabolites in the lesioned striatum of rats pretreated with Lac1, while the effects of a higher dose of Cin were less pronounced (Figure 1B–D).

**Figure 2:**
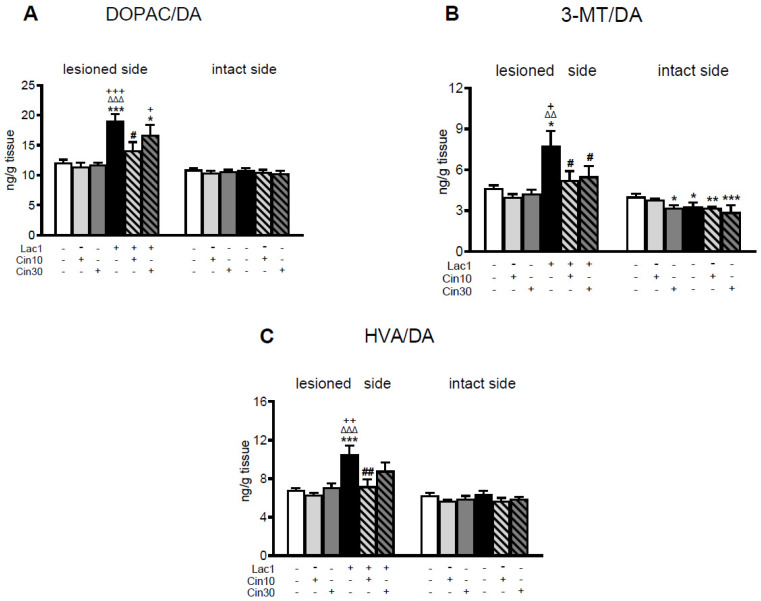
The effect of subchronic i.p. administration of cinnarizine (Cin; 10 or 30 mg/kg) or vehicle (veh) on the rate of DA catabolism in the lesioned and intact striatum of rats injected unilaterally with a proteasome inhibitor, lactacystin (Lac1; 1 μg/2 μL) or vehicle (veh) into the left SNc, assessed as DOPAC-to-DA, 3-MT-to-DA, and HVA-to-DA concentration ratios. The data are presented as the mean ± S.E.M.; the number of rats per each experimental group and the statistical analyses of the given data were the same as in the description in Figure 1. The effects of one-way ANOVA in the lesioned striatum were as follows: (**A**) for DA/DOPAC F_(5,47)_ = 7.701, *p* < 0.0002; (**B**) for 3-MT/DA F_(5,47_) = 3.969, *p* < 0.01; (**C**) for HVA/DA F_(5,47)_ = 5.850, *p* < 0.0005. The effects of this analysis on the intact side for DOPAC/DA and HVA/DA were non-significant; only for 3-MT/DA was its effect significant, F_(5,47)_ = 5.793, *p* < 0.001. Symbols indicate the significance of differences between the tested groups according to the Newman–Keuls post hoc test: * *p* < 0.05, ** *p* < 0.01, *** *p* < 0.001 vs. veh + veh-; ^ΔΔ^ *p* < 0.01, ^ΔΔΔ^ *p* < 0.001 vs. veh + cin10-; ^+^ *p* < 0.05, ^++^ *p* < 0.01, ^+++^ *p* < 0.001 vs. veh + cin30-; ^#^ *p* < 0.05, ^##^ *p* < 0.01 vs. Lac1 + veh-treated groups.

As to DA catabolism, Lac1 evoked an acceleration of the monoamine oxidase (MAO)-dependent intracellular oxidative DA catabolism, catechol-O-methyltransferase (COMT)-dependent extracellular DA catabolism, and total DA catabolism expressed as DOPAC/DA, 3-MT/DA, and HVA/DA catabolic ratios in the lesioned striatum, respectively (Figure 2A–C). Cin at both examined doses partially attenuated the Lac1-enhanced DA catabolism in the lesioned striatum. The effect of a lower dose of Cin on DA catabolism was more strongly pronounced than a higher one (Figure 2A–C).

**Figure 3:**
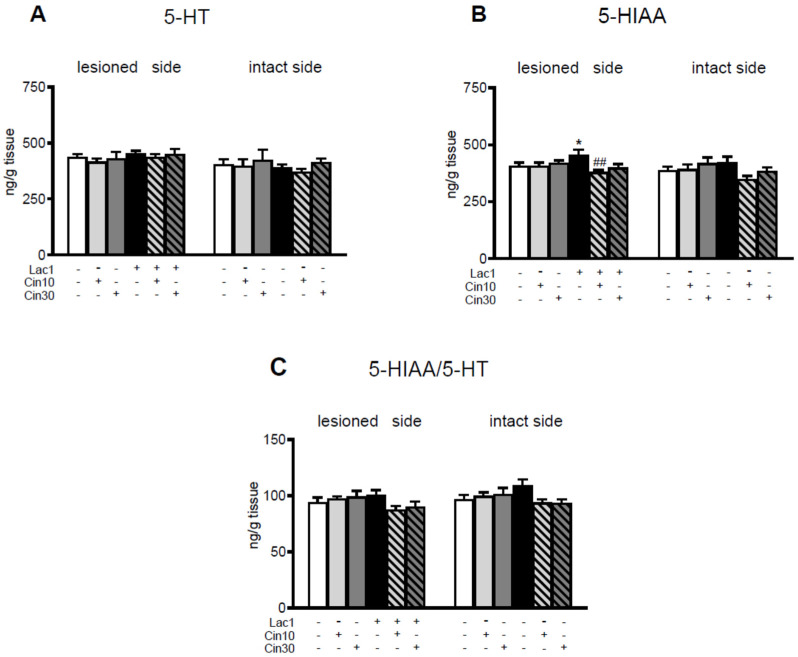
The effect of chronic i.p. administration of cinnarizine (Cin; 10 or 30 mg/kg) or vehicle (veh) on concentrations of serotonin (5-HT), its metabolite 5-hydroxyindoleacetic acid (5-HIAA), and on the rate of 5-HT catabolism assessed as the 5-HIAA-to-5HT concentration ratio in the lesioned and intact striatum of rats injected unilaterally with a proteasome inhibitor, lactacystin (Lac1; 1 μg/2 μL), or vehicle into the left SNc. The data are presented as the mean ± S.E.M.; the number of rats per each experimental group and the statistical analyses of the given data were the same as in the description in Figure 1. Only the effect of one-way ANOVA for 5-HIAA (**B**) in the lesioned striatum was significant, F_(5,47)_ = 3.142, *p* < 0.02, while the remaining analyses for 5-HT (**A**) and 5-HIAA/5-HT (**C**) were insignificant. Symbols indicate the significance of differences between the tested groups according to the Newman–Keuls post hoc test: * *p* < 0.05 vs. veh + veh-; ^##^ *p* < 0.01 vs. Lac1 + veh-treated groups.

Neither unilateral injection of Lac1 into the SNc nor i.p. subchronic administration of Cin in the tested doses affected 5-HT concentrations in the lesioned and intact striatum of the examined groups of rats (Figure 3).

However, in the Lac1 + veh-treated group, a significant increase in the 5-hydroxyindoleacetic acid (5-HIAA) content was observed in the lesioned striatum compared to the veh + veh-treated group, whereas i.p. subchronic administration of 10 mg/kg of Cin reversed the Lac1-induced effect in this brain structure (Figure 3B).

#### 2.1.2. Impact of 10 mg/kg of Cinnarizine on the Tyrosine Hydroxylase (TH) Protein Level in the Substantia Nigra of Lactacystin-Pretreated Rats

**Figure 4:**
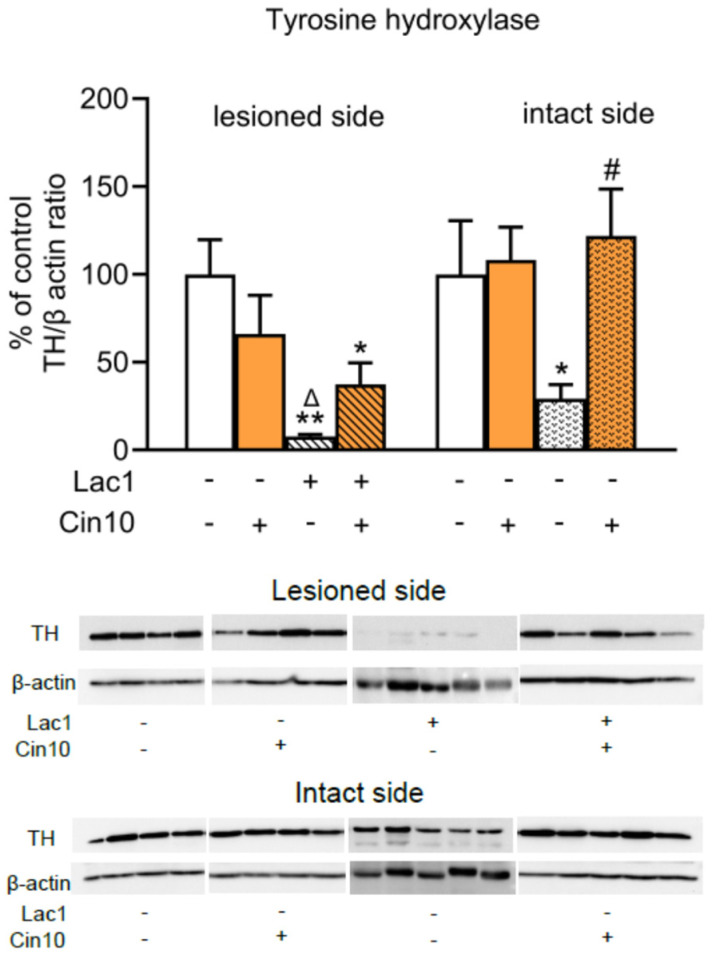
The effect of chronic i.p. administration of 10 mg/kg of cinnarizine (Cin10) or vehicle (veh) on the levels of TH protein in the lesioned and intact SN of rats injected unilaterally with a proteasome inhibitor, lactacystin (Lac1; 1 μg/2 μL), or vehicle into the left SNc. Each bar in the graph represents the mean ± S.E.M. from 4 to 5 lesioned or intact SN samples. Each sample was pooled from tissues from two rats. Rats were killed two hours after the last i.p. doses of Cin10 or vehicle. Levels of TH protein were normalized by calculating the TH/β-actin ratios for each sample. In the control group, the means of normalized TH protein levels in the lesioned and intact SN were taken as 100%. The data shown in the graph are presented as a percentage of control in the veh + veh, veh + Cin10, Lac1 + veh, and Lac1 + Cin10-treated groups. Statistical significance of differences between the examined groups within the lesioned or intact side was calculated using two-way ANOVA, followed (if significant) by the Newman–Keuls post hoc test, * *p* < 0.05, ** *p* < 0.01 vs. the veh + veh- and ^Δ^ *p* < 0.05 vs. the veh + Cin10-; ^#^ *p* < 0.05 vs. Lac1-treated groups. The lower graphs present representative blots of TH protein in the lesioned and intact SN of the examined groups. Supplementary Materials present the raw data taken for Western Blot analysis performed in the lesioned and intact SN of the tested groups.

Western blot analysis of TH protein levels in the lesioned and intact SN was performed in four groups of rats (veh + veh, veh + Cin10, Lac1 + veh, and Lac1 + Cin10) receiving unilaterally a single microinjection of veh or Lac (1 μg/2 μL) into the SNc and i.p. Cin (10 mg/kg) 4 h after surgery and consistently once a day for seven consecutive days (Figure 4).

Two-way ANOVA performed for the TH protein level in the lesioned SN of the tested groups showed a significant effect of microinjection of Lac1 into this structure (F_(1,14)_ = 16.914, *p* < 0.01), a lack of subchronic i.p. treatment with Cin10 (F_(1,14)_ = 0.0196, *p* > 0.05), and a significant interaction between Lac1 and Cin10 (F_(1,14)_ = 4.676, *p* < 0.05) regarding this parameter. Post hoc comparison of the analyzed groups showed that Lac1 drastically reduced the TH protein level in the lesioned SN compared to the veh + veh and veh + Cin10-treated groups (Figure 4). However, in the Lac1 + Cin10-treated rats, the TH protein level, although still significantly lower than in the veh + veh-treated group, was markedly higher than in the Lac1 + veh-treated group. The analogical two-way ANOVA calculated for the TH protein level in the studied groups of intact SN revealed a lack of treatment effect of Lac1 (F_(1,14)_ = 1.632, *p* > 0.05), a significant effect of subchronic i.p. treatment with Cin10 (F_(1,14)_ = 5.078, *p* < 0.05), and no interaction between Lac1 and Cin10 (F_(1,14)_ = 3.560, *p* = 0.0802). The above analysis showed that the dose of 10 mg/kg of Cin maintained the high TH protein levels in the intact SN of both rat groups receiving this drug, veh + Cin10 and lac1 + Cin10. It means that i.p. administered Cin10 efficiently prevented the loss of TH protein induced by Lac1 penetrating from the left lesioned SN side to the right intact side (Figure 4).

#### 2.1.3. Impact of 30 mg/kg of Cinnarizine on the Number of TH-Ir and CV-Stained Neurons in the SN of Lactacystin-Treated Rats

To assess whether cinnarizine (Cin) protects TH-ir and CV-stained neurons in the SN from unilateral Lac1-induced degeneration, a quantitative analysis of the number of these neurons was performed on the lesioned and intact sides.

**Figure 5:**
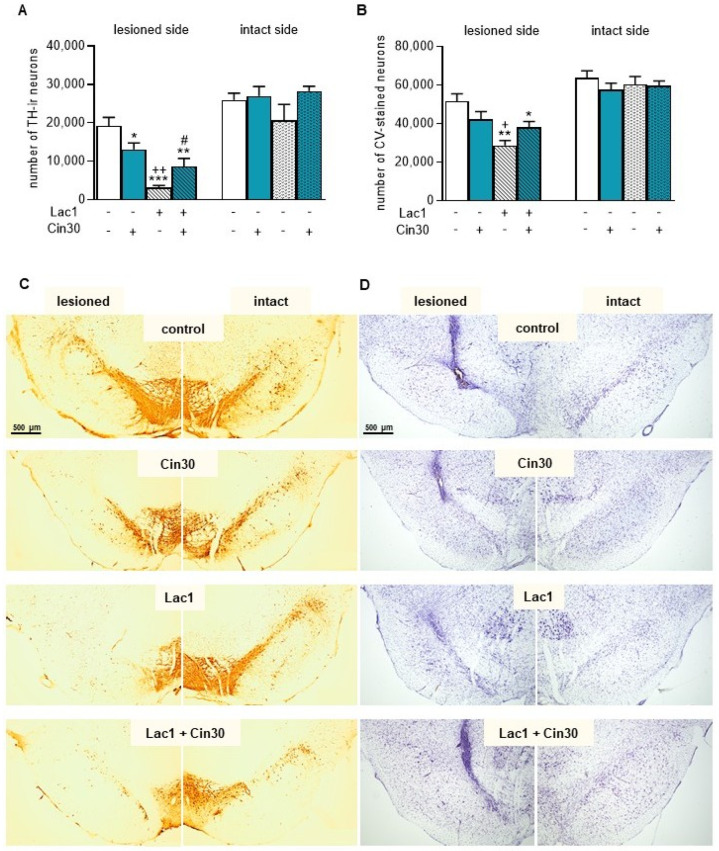
The effect of lactacystin (Lac1) and cinnarizine (Cin30), administered alone or in combination, on the number of TH-immunoreactive (TH-ir) neurons (**A**,**C**) and CV-stained neurons (**B**,**D**) in the lesioned and intact SN of rats. Lac at a dose of 1 μg/2 μL (Lac1) was unilaterally injected into the left SNc. Cin at 30 mg/kg (Cin30) was administered i.p. once daily for seven days. (**A**,**B**) graphs illustrating the stereological quantification of TH-ir (**A**) and CV-stained neurons (**B**) in the SN of rats after treatment with Lac1, Cin30, or their combination. (**C**,**D**) representative microphotographs of coronal sections showing TH-ir (**C**) and CV-staining neurons (**D**) in the SN of rats across the examined group. Glial scarring and tissue destruction (in **D**) are observed at the injection sites. Calibration bars for (**C**,**D**) are 500 μm. The data are presented as the mean ± S.E.M.; the number of rats for each experimental group was as follows: veh + veh (control) = 6, veh + Cin30 = 6, Lac1 + veh = 5, and Lac1 + Cin30 = 6. Statistical significance of differences between the studied groups in the lesioned and intact sides was calculated using the two-way ANOVA, followed (if significant) by the Newman-Keuls post hoc test: *** *p* < 0.001, ** *p* < 0.01, * *p* < 0.05 vs. veh + veh-; ^++^ *p* < 0.01, ^+^ *p* < 0.05 vs. veh + Cin30-; ^#^ *p* < 0.05 vs. Lac1 + veh-treated groups.

Two-way ANOVA for the number of TH-ir neurons in the lesioned SN revealed a significant effect of Lac1 injection (F_(1,19)_ = 31.270, *p* < 0.0001), no impact of subchronic i.p. treatment with Cin30 (F_(1,19)_ = 0.034, *p* > 0.05), and a significant interaction between Lac1 and Cin30 (F_(1,19)_ = 10.162, *p* < 0.005). Post hoc analysis confirmed that Lac1 drastically reduced the number of TH-ir neurons in the lesioned SN (Figure 5A). Interestingly, i.p. treatment with Cin30 alone in rats receiving vehicle into the SN (veh + Cin30 group) led to a slight reduction in the number of TH-ir neurons. However, in Lac1-treated rats, Cin30 partially protected TH-ir neurons from degeneration (Figure 5A). Nevertheless, the number of TH-ir neurons in this group, although significantly higher than in the Lac1 + veh-treated group, was still lower than in the veh + veh-treated control.

Similarly, two-way ANOVA for the number of CV-stained neurons in the lesioned SN revealed a significant effect of Lac1 injection (F_(1,19)_ = 15.161, *p* < 0.001), no significant impact of subchronic i.p. treatment with Cin30 (F_(1,19)_ = 0.0001, *p* > 0.05), and a significant interaction between the Lac1 and Cin30 (F_(1,19)_ = 7.284, *p* < 0.05; Figure 5B). Post hoc comparison demonstrated that an intranigral Lac1 administration significantly reduced the number of CV-stained neurons in the lesioned SN compared to the veh + veh- and veh + Cin30-treated groups. Subchronic i.p. Cin30 administration showed a trend toward reversing the Lac1-induced effect (*p* = 0.07; Figure 5B).

In the intact SN, two-way ANOVA for TH-ir or CV-stained neurons showed no significant treatment effects of Lac1 and Cin30, indicating no significant differences in the number of these neurons (Figure 5A,B). Figure 5C,D shows representative pictures of TH-ir and CV-stained neurons, respectively, in the SN of rats after administration of the tested compounds or their solvents.

### 2.2. Effects of the In Vitro Study

#### 2.2.1. Effect of Cinnarizine on Lactacystin- and Rotenone-Induced Cell Damage in SH-SY5Y Cells

**Figure 6:**
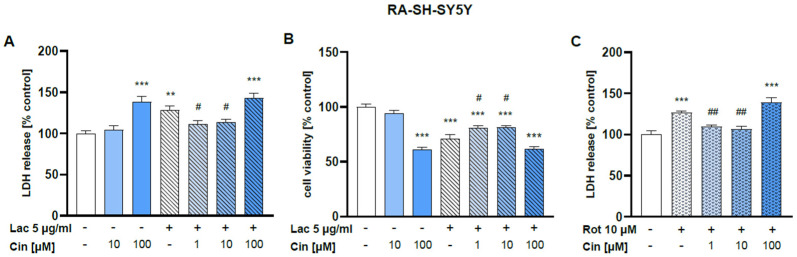
Neuroprotective effects of cinnarizine (Cin) against lactacystin (Lac) or rotenone (Rot)-evoked cell damage in SH-SY5Y cells. The cells were treated with Cin (10 or 100 μM) alone or Cin (1–100 μM) combined with Lac (5 μg/mL) for 48 h (**A**,**B**) or Cin (1–100 μM) combined with Rot (10 μM) for 24 h (**C**). Cytotoxicity was measured by the LDH release assay (**A**,**C**), whereas cell viability was assessed by the MTT reduction test (**B**). The data were normalized to control and are presented as a mean ± S.E.M. for two independent experiments with five replicates, *** *p* < 0.001, ** *p* < 0.01 vs. control group, ^##^ *p* < 0.01, and ^#^ *p* < 0.05 vs. Lac or Rot group.

Cin at a concentration of 100 μM but not 10 μM was found to be toxic for SH-SY5Y as confirmed by lactate dehydrogenase (LDH) release and 3-[4,5-dimethylthylthiazol-2-yl]-2,5-diphenyltetrazolium bromide (MTT) reduction assays (Figure 6A,B).

The concentration of 5 μg/mL of Lac evoked about a 30% reduction in cell viability after 48 h of treatment (Figure 6A), which was partially prevented by 1 or 10 μM Cin (Figure 6A). These protective effects of Cin were also confirmed by cytotoxicity assay, where the Lac-induced LDH release was partially attenuated by 1 and 10 μM Cin (Figure 6B). Cin at 1 and 10 μM, but not at 100 μM, was also protective against the rotenone-evoked SH-SY5Y cell death, as evidenced by the LDH release assay after 24 h of treatment (Figure 6C).

#### 2.2.2. Effect of Cinnarizine on Lactacystin- or Rotenone-Induced Cell Damage in Primary Cortical Neurons

**Figure 7:**
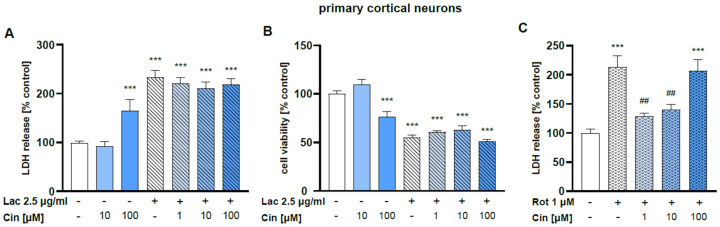
The effects of cinnarizine (Cin) against the lactacystin (Lac) or rotenone (Rot)-evoked cell damage in primary cortical neurons. The 7 DIV cortical neurons were treated with Cin (10 or 100 μM) alone or Cin (1–100 μM) combined with Lac (Lac 2.5; 2.5 μg/mL) for 48 h (**A**,**B**) or Cin (1–100 μM) combined with Rot (1 μM) for 24 h (**C**). Cytotoxicity was measured by the LDH release assay (**A**,**C**), whereas cell viability was assessed by the MTT reduction test (**B**). The data were normalized to control and are presented as a mean ± S.E.M. for two independent experiments with five replicates, *** *p* < 0.001 vs. control group, ^##^ *p* < 0.01 vs. Rot group.

Cin at a concentration of 100 μM but not 10 μM was found to be toxic for primary cortical neurons, as confirmed by LDH release and MTT reduction assays (Figure 7A,B).

The concentration of 2.5 μg/mL of Lac evoked about a 45% reduction in cell viability after 48 hr of treatment, which was not changed by co-treatment with Cin (1–100 μM) (Figure 7A). Similarly, Cin (1–100 μM) also did not affect the Lac-evoked LDH release (Figure 7B), which further evidences its lack of protection in this type of cell damage. However, Cin at 1 and 10 μM, but not at 100 μM, significantly attenuated the Rot-induced cell death in primary cortical neurons, as evidenced by the LDH release assay after 24 h of treatment (Figure 7C).

## 3. Discussion

Biochemical and histological studies performed in the unilateral Lac1-induced rat model of PD showed for the first time that subchronic i.p. treatment with the dual T- and L-type voltage-gated Ca^2+^ channel blocker, Cin, partially prevented both the decline in tissue DA content in the striatum and the loss of the TH-ir neurons in the SN. In general, Ca^2+^ channel antagonists (mainly dihydropyridine derivatives) belonging to various pharmacological classes affect DA metabolism differently, and these changes, if they occur, are regionally specific [79,80,81,82]. However, the influence of Cin, representing diphenylpiperazines, on DA metabolism, despite many controversies regarding its induction of extrapyramidal symptoms [83,84,85], has not yet been studied in animal models of PD.

Regarding the impact of Cin on tissue DA and its metabolite concentrations in the lesioned striatum of the Lac1-pretreated group, 10 mg/kg of Cin was more effective, significantly increasing its contents 2 h after its last subchronic dose. In comparison, 30 mg/kg of Cin was less effective, eliciting only an increasing trend in the tissue concentrations of DA and its metabolites, DOPAC and HVA, in the lesioned striatum vs. the values of these parameters in the Lac1 + veh-treated group. Regarding DA catabolism, unilateral administration of Lac1 accelerated both the MAO-dependent intracellular oxidative DA catabolism and COMT-dependent extracellular DA catabolism, as well as total DA catabolism measured as DOPAC/DA, 3-MT/DA, and HVA/DA catabolic ratios in the lesioned striatum, respectively. A low dose of Cin very effectively reduced the values of these indicators, whereas the effectiveness of a higher dose was clearly weaker. The more beneficial effect of 10 mg/kg Cin on DA catabolism compared to 30 mg/kg of this drug may be primarily due to a stronger inhibition of intracellular oxidative DA catabolism, expressed as the DOPAC/DA metabolic index. Its value was significantly reduced compared to the Lac1 + veh group. In contrast, after administration of the higher dose of Cin, only a slight downward trend was observed for this parameter compared to the Lac1 + veh group. In general, during the intracellular DA catabolism catalyzed by MAO in DA neurons, in addition to DOPAC, hydrogen peroxide is produced, which in the presence of iron (Fe^2+^) ions can become a source of the highly reactive hydroxyl radical. Therefore, from the perspective of neuroprotective effects, the lower dose of Cin appears to be more optimal than the higher one.

Cinnarizine is not only a VGCC blocker but also a dopamine D2 receptor antagonist of low-to-moderate affinity (Ki = 13.2 nM), falling between olanzapine (Ki = 3.7 nM) and clozapine (Ki = 40 nM) in terms of D2 binding. This classification is based on in vivo receptor occupancy and in vitro affinity [83,86]. Therefore, Cin shares the main mechanism of action characteristic of atypical antipsychotics [87,88]. Furthermore, Cin exerted a potent antagonistic effect on the 5-HT2 receptor in vivo (Ki = 0.32 nM) [89], similar to atypical antipsychotics such as olanzapine and clozapine [90]. Therefore, we assume that in our study, in addition to the blockade of Ca^2+^ channels, Cin receptor activity may play a significant role in DA metabolism. Thus, we postulate that the blockade of presynaptic DA D2 receptors in the Lac1-pretreated rats with a low dose of Cin may eliminate the tonic inhibitory action of DA on its synthesis; in turn, the blockade of postsynaptic DA D2 receptors, by triggering the striato-nigral feedback loop, may affect TH activity and thereby increasing tissue DA levels in the lesioned striatum, despite a significantly lower TH protein concentration in the tested groups compared to the veh + veh control group. On the other hand, blockade of postsynaptic DA D2 receptors in the striatum by typical antipsychotic drugs (e.g., haloperidol) is associated with the risk of extrapyramidal symptoms. Hence, the antagonistic effect of Cin on postsynaptic DA D2 receptors may theoretically limit its use in the treatment of PD. However, we hypothesize that the pharmacological profile of Cin, which resembles that of atypical antipsychotics, and the low dose range of this drug used in our study may reduce the risk of these symptoms. Consistent with these assumptions, behavioral studies have shown that Cin at a dose of 20 mg/kg counteracted both amphetamine- and MK-801-induced hyperlocomotion but did not affect spontaneous locomotor activity in mice [84]. Moreover, this dose of Cin also did not induce catalepsy, with only its high doses (60 and 180 mg/kg) eliciting mild catalepsy [84]. Therefore, it seems likely that in PD therapy, a low dose of Cin administered in combination with L-DOPA could have a beneficial effect on motor functions by reducing hyperlocomotion and on cognition by preventing L-DOPA-induced hallucinations.

As to DA catabolism, in presynaptic dopaminergic terminals in the striatum, Ca^2+^ influx via VGCC triggers the release of vesicular DA into the synaptic cleft, where it is metabolized by COMT, forming 3-MT, or taken up by the dopamine transporter (DAT) localized on these terminals back to the cytoplasm [25,91]. Hence, the presence of 3-MT in the extracellular space is considered an indicator of DA release and a measure of a functional synaptic pool of this neurotransmitter [92]. In our study, the comparable striatal 3-MT levels in the striatum of control groups veh + veh and veh + Cin10 indicate that Cin at a dose of 10 mg/kg did not affect the Ca^2+^-dependent DA release on both sides of this brain structure. The effect of Cin in the above-presented control groups is in agreement with findings reported in [93], where flunarizine, belonging to the same chemical group as Cin and acting as a T-type channel blocker, administered at a concentration of 10 µM directly into the striatum, did not affect DA release or its metabolism in this brain structure. However, in our study, the increased 3-MT level in the lesioned striatum of the Lac1 group receiving i.p., subchronically 10 mg/kg of Cin, compared to the 3-MT content in the Lac1 group receiving the veh, shows that the tested Cin dose was effective in preventing the reduction of the striatal functional pool of DA in the used rat model of PD.

On the other hand, comparable levels of 3-MT in the lesioned striatum of the Lac1 + veh and Lac1 + Cin30 rat groups, as well as a decreasing tendency in the 3-MT content in the veh + Cin30 group, show that a higher dose of Cin may reduce the Ca^2 +^ -dependent DA release in the striatum. The present analysis of DA catabolism from the unilateral Lac1-induced rat model of PD subchronically treated with two different doses of Cin shows that its low dose (10 mg/kg) protects the dopaminergic terminals in the lesioned striatum against the loss of synaptic DA more distinctly than the higher one (30 mg/kg). Thus, we assume that the reducing effect of the higher dose of Cin on DA release may result from its blockade of L-type Ca^2+^ channels in the striatum.

In line with the above assumption, blockade of L-type VGCCs by the selective dihydropiridine derivative nimodipine, in the MPTP-induced PD model in mice and non-human primates, resulted in a drastic depletion of DA in the caudate-putamen, despite almost complete protection of TH-ir neurons in the SN [50,52,53]. Consistent with the above data, previous studies demonstrated that nimodipine decreases tissue DA and 3-MT levels in the striatum and reduces DA synthesis, as assessed by L-DOPA accumulation [94,95]. Furthermore, a previous study using microdialysis demonstrated that other L-type Ca^2+^ channel blockers, such as verapamil (1–300 µM) and nicardipine (1–100 µM), administered intrastriatally, decreased DA release [93]. Finally, the dihydropyridine antagonist with the highest affinity for Cav1.3 L-type channels, istradipine, protects cell bodies in the SN against MPTP [23,50]. However, in the MPTP model of PD, istradipine, similar to nimodipine, induces a decrease in DA concentration in the striatal synaptic pool [36]. The neuroprotective effect of istradipine was also observed in the 6-OHDA-induced PD rat model, but only when a low dose of this neurotoxin was injected intrastriatally [54].

Regarding DA release in the striatum, it has recently been shown that Ca^2+^ influx via L-VGCC into dopaminergic axonal terminals is critical for its release, especially in the dorsolateral part of the striatum [36,96]; hence, inhibition of these channels may reduce DA release in this region of the striatum. On the other hand, Ca^2+^ influx via L-VGCC into the DA neuronal bodies in the SN generates metabolic stress that promotes degeneration [51], while inhibition of these channels is associated with neuroprotection. The data presented above suggest that selective L-VGCC blockers such as nimodipine or dual L- and T-type with preferential activity toward L-type Ca^2+^ channels, such as istradipine, may protect nigrostriatal DA neurons from degeneration at the level of nigral cell bodies but did not prevent the decrease in the synaptic striatal pool of DA, whereas drugs preferentially blocking T-VGCC, such as Cin used in our study, administered at low doses, can protect them at both the nigral cell bodies level and at the biochemical level, preventing the decline in the content of synaptic DA in the striatum.

Regarding the most characteristic marker of PD, i.e., the TH protein level in the SN, the unilateral intranigral injection of Lac1 significantly decreased its content in the lesioned and intact sides of this structure. The effect of Lac1 on the TH protein level on the lesioned side was consistent with the decrease in the number of TH-ir neurons in the SN. However, on the unlesioned side, despite a significant decline in the TH protein level, no changes were observed in the number of TH- and CV-stained neurons, as assessed quantitatively by stereological counting, compared to the control group. Notably, in our previous study [18], double staining of the SN to visualize TH protein and neuronal cell bodies (CV) showed that, in neurons of the unlesioned side of the SNc, which morphologically correspond to DA neurons, TH protein disappeared following intranigral Lac1 administration. The latter effect suggests that, as a result of the penetration of a small amount of Lac1 from the lesioned to the unlesioned side, TH protein synthesis was inhibited. In the present study, subchronic treatment with 10 mg/kg partially reversed the deleterious effect of Lac1 on the TH protein level in the lesioned SN. Simultaneously, the TH protein level in the intact SN fully returned to control values.

Initially, when proteasome inhibitors were proposed as model compounds to recapitulate Lewy body-like intracytoplasmic inclusions [97,98,99], it was assumed that protein synthesis remained unchanged under conditions of proteasome inhibition. However, it was later found that inhibition of the proteasome function profoundly and preferentially suppressed the synthesis of many proteins belonging to the synaptic pool [100,101,102]. Furthermore, short-term inhibition of proteasome function and subsequent decline in protein synthesis are reversible and may benefit cells because they prevent the potentially harmful accumulation of proteins. The results of our study seem to confirm the involvement of Lac1 in the inhibition of TH protein synthesis, because Cin at a dose of 10 mg/kg, and not 30 mg/kg, administered i.p., 4 hr after intranigral injection of Lac1 and then for the next seven days, was able to partially prevent the decline in this protein content in the lesioned SN and entirely in the unlesioned side. On the other hand, long-term inhibition of proteasome activity and protein synthesis can lead to a deficiency of many essential proteins and other cellular components, ultimately promoting the development of neuropathological abnormalities and neuronal death [100,101]. The results presented above clearly indicate that a low dose of Cin may effectively restore protein synthesis, which is impaired by short-term inhibition of proteasome function.

Regarding the effect of proteasome inhibitors on cytosolic Ca^2+^ concentration, studies in various cell culture models have shown that, in most cases, they increase cytosolic Ca^2+^ levels [103,104,105]. However, one study reported a decrease [34]. The reason for the discrepancy in such action of proteasome inhibitors requires explanation. Generally, average Ca^2+^ concentrations in the neuronal cytosol are in the nM range, in contrast to the mM concentrations in the extracellular space and the endoplasmic reticulum (ER). Most Ca^2+^ ions flowing into neuronal cytoplasm are sequestered by the ER and mitochondria or bound to Ca-binding proteins [106,107,108].

On the other hand, Ca^2+^ is also released from the ER via ryanodine (RyRs) and inositol 1,4,5-triphosphate (IP3) receptors and from mitochondria via the mitochondrial sodium-calcium exchanger [38,109,110]. Hence, an increase in intracellular Ca^2+^ levels in mature DA neurons, resulting from excessive influx from the extracellular space and release from intracellular storage organelles, can compromise Ca^2+^ regulatory mechanisms [106], initiating pathological changes. In contrast, compounds that affect both processes can protect DA neurons by normalizing intracellular Ca^2+^ homeostasis. Assuming an increase in cytosolic Ca^2+^ concentration in DA neurons following Lac1 administration into the SNc, Cin, as a dual blocker of voltage-gated T- and L-type Ca^2+^ channels, administered at low doses, preferentially inhibited Ca^2+^ influx through T-type channels and less effectively through L-type channels. Since in the SNc, T-type Ca^2+^ channels are predominantly located on Calb-negative DA neurons with a very low level of this protein [69], we can speculate that this drug may effectively protect these neurons, as they are more sensitive to the increase in the cytosolic Ca^2+^ content than Calb-positive DA neurons containing a high level of Calb protein. Discussing other neuroprotective effects of Cin in the nervous system, systemic administration of Cin for 1 week has been shown to prevent neuronal cell death following facial nerve injury, and repeated Cin treatment for 1 month promotes regeneration of the facial nerve [111].

Interestingly, Cin, besides blocking T- and L-type Ca^2+^ channels, can influence the number of intracellular mechanisms. It is worth mentioning the protective effect exerted by this drug on mitochondrial function [112]. The toxic effect of a high Ca^2+^ concentration (25 μM) on mitochondrial function is based on increased transition permeability of their membrane to this ion, NAD(P)H oxidation, and ultimately, the collapse of the mitochondrial membrane. Under such conditions, low concentrations of Cin (<50 μM) inhibited mitochondrial Ca^2+^ overload and NAD(P)H oxidation and restored the mitochondrial membrane potential decreased by a high Ca^2+^ concentration. However, at higher concentrations (>50 μM), Cin was able to induce mitochondrial swelling by itself, mitochondrial NAD(P)H was oxidized, and the membrane potential collapsed.

The neuroprotective properties of Cin were also evaluated in cultures of human neuroblastoma SH-SY5Y cells and primary cortical neurons. The SH-SY5Y cell line possesses a dopaminergic phenotype and, therefore, is widely used as an in vitro model to test potential neuroprotective compounds that could be used in the therapy of PD [113,114,115,116,117]. In our study, low concentrations of Cin (1 and 10 μM) partially protected SH-SY5Y cells from Lac toxicity, as assessed by LDH release and MTT reduction assays. The latter effects are consistent with the neuroprotective profile of a low dose of Cin in the Lac1-induced rat model of PD presented in the first part of our study. However, in the culture of cortical neurons, Cin, administered at the same concentration ranges as in SH-SY5Y cell culture, was ineffective against Lac toxicity in both assays.

In addition to impaired proteasome function in the SN of PD patients, a reduction of mitochondrial complex I activity has also been reported [118,119]. An inhibitor of mitochondrial complex I, rotenone, was used as a model substance to induce mitochondrial dysfunction in the studied cell cultures [120,121,122]. In our study, administration of Cin at low concentrations (1 and 10 μM) to cultures of SH-SY5Y cells and cortical neurons protects them against Rot-induced toxicity as evaluated in the LDH release assay.

Moreover, the neuroprotective effect of Cin against Rot-induced toxicity was recently confirmed in cultures of induced pluripotent stem cells (iPSCs) derived from patients with the familial form of PD, specifically those carrying a mutation in the PARKIN PARK2 gene, which is involved in mitochondrial homeostasis and stress responses [77]. Cin effectively protected iPSC-derived dopaminergic (DA) neurons (PARK2-DA) from Rot toxicity in that study [77].

Tabata et al. [77] also demonstrated that PARK2-DA neurons exhibit increased expression of T-type Ca^2+^ channels and elevated intracellular Ca^2+^ concentrations. Hence, it was reasonable to assume that the selective vulnerability of PARK2-DA neurons to Rot-induced stress was attributable to the dysregulation of intracellular Ca^2+^ homeostasis via T-type Ca^2+^ channels. These findings suggest a crucial role for T-type Ca^2+^ channels in the pathogenesis of PD. Therefore, blocking these channels may offer significantly greater therapeutic potential than inhibiting L-type Ca^2+^ channels, particularly those of the Cav1.3 subtype, as previously postulated in numerous studies. In light of this, future neuroprotective strategies for PD should focus on identifying selective T-type Ca^2+^ channel blockers and evaluating their efficacy in various PD models.

## 4. Materials and Methods

### 4.1. Animals

The study used adult 3-month-old male Wistar rats (Charles River, Sulzfeld, Germany) with an initial body weight ranging between 280 and 320 g and pregnant CD1 mice (Charles River, Sulzfeld, Germany). Rats were kept under standard laboratory conditions in groups of 5 per large cage, at a temperature of 22 ± 2 °C, a humidity of 45–50%, and under an artificial 12/12 h light/dark cycle, with free access to standard laboratory food and tap water. Pregnant CD1 mice were kept individually in a small cage under the same standard conditions.

All experimental procedures referring to the in vivo study and those for generating primary neuronal cell culture for the in vitro study were conducted following the Act on the Protection of Animals Used for Scientific or Educational Purposes of 21 January 2005 and in compliance with the Directive of the European Parliament and of the Council of Europe 2010/63/EU of 22 September 2010 on the protection of laboratory animals. The Bioethics Commission of the Institute of Pharmacology, Polish Academy of Sciences, approved the experimental protocols in Kraków, Poland (permission no. 658/2009 of June 2009). All efforts were made to reduce the number of rats used in experiments to limit their suffering while simultaneously ensuring that the results obtained were statistically reliable (3R policy).

### 4.2. In Vivo Study

#### 4.2.1. Surgery

This procedure followed the previously described method [18,19]. Briefly, the rats scheduled for surgery were lightly anesthetized with pentobarbital (Vetbutal, 30 mg/kg i.p., Biowet Puławy Sp. z o.o., Puławy, Poland) and then placed in a stereotaxic apparatus. A stainless-steel needle (0.28 mm outer diameter) was unilaterally inserted through a small hole in the skull, with its tip positioned in the left SNc according to the coordinates of the Paxinos and Watson atlas [123]: A = −5.2 mm, L = 1.8–2.4 mm, H = 7.6–8.2 mm. Through this cannula, lactacystin (Lac; Sigma-Aldrich Chemie GmbH, Steinheim, Germany) dissolved in redistilled water was injected at 1 μg in a volume of 2 μL into the left SNc at a flow rate of 0.5 μL/min using a Hamilton microsyringe. Control rats received a vehicle(veh) instead of Lac. The cannula was left in place for at least 10 min to allow diffusion. After surgery, animals were placed in recovery cages and monitored until fully awake, at which point they were returned to their home cages.

#### 4.2.2. Experimental Groups and Drug Administration

The experiments were carried out on six groups of rats; three received Lac (1 μg/2 μL), and the remaining three received veh instead, unilaterally into the left SNc. Of the three groups of rats receiving Lac, the control group was administered i.p. veh, and the other two received cinnarizine (Cin; Cat. No. C5270; Sigma-Aldrich Chemie GmbH, Steinheim, Germany) at doses of 10 or 30 mg/kg. Similarly, the three groups of rats receiving the vehicle unilaterally into the left SNc were i.p. administered veh and Cin at the tested doses. In all examined groups, the first i.p. doses of Cin or veh were administered 4 h after surgery and once daily for seven subsequent days.

Two hours after the last doses of veh or Cin (10 and 30 mg/kg), the rats were sacrificed by decapitation, and the brains were extracted from the skulls. Each brain was cut into anterior and posterior parts. Then, from the anterior parts, the left- and right-sided striata were dissected on an ice-chilled glass plate to be used to investigate the metabolism of dopamine (DA) and serotonin (5-HT) in these brain structures. Then the striatal tissue samples were stored at −80 °C until the HPLC procedure was applied. The left and right SN dissected from the posterior parts of the rat brain from groups receiving veh or Lac unilaterally into the left SNc (1 μg/2 μL) and treated i.p. with veh or 10 mg/kg of Cin were used for Western blot analysis of tyrosine hydroxylase (TH) protein levels. In turn, in the groups of rats receiving Lac unilaterally into the left SNc and i.p. veh or 30 mg/kg of Cin, the posterior parts of the brain, including the SN, were subjected to histological examination.

#### 4.2.3. Dopamine and Serotonin Metabolism in the Striatal Homogenates

Dopamine (DA), serotonin (5-HT), and their metabolites—homovanilic acid (HVA), 3,4-dihydroxyphenylacetic acid (DOPAC), 3-methoxytyramine (3-MT), and 5-hydroxyindoleacetic acid (5-HIAA)—were assayed in striatal homogenates, separately for the left (lesioned) and right (intact) sides, by reverse-phase high-performance liquid chromatography (HPLC) with coulometric detection. Tissue samples were weighed and homogenized in ice-cold 0.1 M perchloric acid containing 0.05 mM ascorbic acid. After centrifugation (10,000× *g*, 15 min), the supernatants were filtered through 0.2 μm cellulose filters (Alltech Associates Inc., Deerfield, IL, USA) and injected into an HPLC system, which consisted of a P680 pump, an ASI-100 autosampler, and a thermostated column compartment TCC-100 (Dionex, Germering, Germany) equipped with a Hypersil C18 column (100 × 3.0 mm i.d., three μm particle size) fitted with a 10 × 3 mm precolumn (Thermo Fisher Scientific Inc., Waltham, MA, USA). Detection was performed using a Coulochem III detector (ESA Inc., Chelmsford, MA, USA) equipped with a guard cell (ESA 5020, electrode potential set at 600 mV) and a dual electrochemical analytic cell (ESA 5010, applied potential E1 = −200 mV, E2 = 300 mV). The mobile phase consisted of 35 mM citrate/47 mM disodium phosphate buffer (pH 4.2), supplemented with 0.25 mM of EDTA, 0.25 mM of sodium 1-octanesulfonic acid sodium salt, 2.4% methanol, and 1.3% acetonitrile. Temperatures of the analytical cell and the column were maintained at 30 °C, and the flow rate was maintained at 0.8 mL/min. DA, 5-HT, and their metabolites were quantified by peak area comparisons of the tested samples with freshly prepared standards, run on the analysis day. The approximate retention times of the measured substances were as follows: for DOPAC = 4.26, DA = 5.31, 5-HIAA = 7.98, HVA = 12.53, and 5-HT = 14.2 min. Data were collected and analyzed using Chromeleon 6.8 software (Dionex, Germering, Germany).

#### 4.2.4. Western Blots of Tyrosine Hydroxylase (TH) Protein in the Substantia Nigra

Left and right substantia nigra originating from groups of rats received Lac (1 μg/2 μL) or veh unilaterally into the left SNc and additionally treated i.p. with Cin at a dose of 10 mg/kg or veh were used for Western blot analysis of TH protein levels. Individual tissue samples were weighed, homogenized on ice in 20 volumes of RIPA buffer containing a mixture of protease inhibitors (Pierce, *Appleton, WI, USA*), denatured for 10 min at 95 °C, and centrifuged for 10 min at 10,000× *g* at 4 °C. Protein concentration in the supernatants was determined using a bicinchoninic acid protein assay kit (Pierce, Appleton, WI, USA). Afterwards, the samples containing 10 μg of total protein were fractionated by 10% sodium dodecyl sulfate–polyacrylamide gel electrophoresis (SDS-PAGE), as described previously by Laemmli [124], and processed to detect TH. Proteins from resolved gels were then transferred to nitrocellulose membranes (Sigma-Aldrich, St. Louis, MO, USA). Nonspecific binding sites were blocked for one h at room temperature (RT) by a 3% BSA in Tris-buffered saline with a 0.5% Tween 20 (TBS-T) and incubated for two hours with a mouse monoclonal anti-TH antibody (MAB5280, Millipore, Burlington, MA, USA) diluted 1:4000 in 1% BSA in TBS-T at RT. After four subsequent washes in TBS-T, membranes were processed according to the standard BM Chemiluminescence Western Blotting Kit protocol (Roche Applied Science, Penzberg, Germany). Following immunoblot visualization, membranes were blocked with 5% non-fat dry milk in TBS for 10 min at RT and dried on absorbent filter paper. Afterwards, blots were erased in 62.5 mM TrisCl pH 6.8, 2% SDS, and 100 mM 2-mercaptoethanol for 30 min at 50 °C, washed twice with TBS, and blocked overnight with 5% BSA in TBS at 4 °C. As a control for protein level normalization, erased blots were processed with mouse monoclonal anti-β-actin antibody (A5441, Sigma-Aldrich, St. Louis, MO, USA) at a dilution 1:8000, as described above.

The amounts of protein per lane and antibody concentrations were optimized in pilot studies so that at least threefold differences in protein content were linearly reflected on immunoblots. Results are presented as a percentage of the control of the analyzed protein: β-actin ratio ± S.E.M.

#### 4.2.5. Histological Analysis

##### Tissue Collection

The posterior parts of rat brains (mesencephalon and pons), which were collected from the groups receiving unilaterally into the SNc Lac (1 μg/2 μL) or the same volume of veh, and additionally repeatedly treated i.p. with 30 mg/kg Cin or veh, were designed for histological analysis. Then these parts of the brains were fixed in a cold, buffered 4% paraformaldehyde for at least 7 days at 4 °C, immersed in a buffered 20% sucrose solution in PBS, and stored at 4 °C for at least 7 days. Next, the posterior parts of the brains were frozen on dry ice and sectioned into 30-μm-thick frontal slices using a freezing microtome (Reichert (N 17169), Vienna, Austria). Free-floating sections at levels containing SN (AP = −4.8 to −6.30 mm from bregma), according to Paxinos and Watson [123], were collected in 0.01 M PBS, with every sixth section used for histological staining, including immunohistochemistry for TH and Nissel (cresyl violet) staining.

##### TH Immunocytochemistry and Cresyl Violet (CV) Staining in the SN

Immunohistochemical staining was performed to visualize TH-ir neurons as previously described [18,125]. Briefly, the sections were blocked with 5% normal horse serum (NHS; Vector Laboratories, Newark, CA, USA) at room temperature (RT) for 30 min. Next, the sections were incubated for 48 h at 4 °C in a primary mouse monoclonal anti-TH antibody (MAB5280, Millipore, Billerica, MA, USA), diluted at 1:3000 in PBS containing 0.2% Triton™ X-100 (PBS-TX-100; Sigma-Aldrich, St. Louis, MO, USA) and 3% NHS. After that, the sections were washed in PBS and incubated for 30 min at RT in the secondary antibody, such as biotinylated anti-mouse IgG (1:200, Vector Laboratories, Newark, CA, USA), diluted in 0.2% PBS-TX-100 and 3% NHS. Next, the sections were processed by an avidin-biotin-peroxidase complex method using the Vectastain Elite ABC-HRP Kit, peroxidase (Vector Laboratories, Newark, CA, USA), and diaminobenzidine (DAB) (Vector Laboratories, Newark, CA, USA) as a chromogen. The stained sections were mounted on slides, dried, dehydrated, cleared in xylene, cover-slipped with Permount, and prepared for microscopic analysis.

Additionally, to visualize all neuronal cells in the entire SN, sections adjacent to those labeled with the TH antibody were stained with 1% cresyl violet (CV) using the Nissl method, as previously described [126].

##### Stereology Microscopic Analysis

Stereological counting of TH-ir and CV-stained neurons in the whole SN (pars compacta and reticulata) on both sides of the brain was performed using a microscope (Leica DMLB; Leica Microsystems GmbH, Wetzlar, Germany) equipped with a projecting camera (Basler Vision Technologies, Ahrensburg, Germany) and a microscope stage connected to an *xyz* stepper (PRIOR ProScan, Cambridge, UK) controlled by a computer using new-CAST software (Visiopharm A/S, Hørsholm, Denmark) as previously described [18]. The total number of TH-ir and CV-stained neurons was counted under a magnification of ×63 using randomized meander sampling and the optical dissector method, following previously described mathematical formulas [18]. The counting frame had an area of 3321.1 μm^2^, covering 12% of the screen area for analysis. The dissector height was set at 15 μm, and the sampling grid dimensions were 257.33 μm × 257.33 μm (equivalent to 66,218.7 μm^2^).

### 4.3. In Vitro Experiments

#### 4.3.1. Chemicals

Dulbecco’s modified Eagle medium (DMEM), 0.25% trypsin/EDTA solution, penicillin-streptomycin solution, heat-inactivated fetal bovine serum (FBS), Neurobasal A medium, and supplement B27 without antioxidants were from Gibco (Invitrogen, Paisley, UK). The Cytotoxicity Detection Kit was from Roche Diagnostics (Mannheim, Germany). All other reagents were from Sigma-Aldrich Chemie GmbH (Steinheim, Germany).

#### 4.3.2. SH-SY5Y Cell Culture

Human neuroblastoma SH-SY5Y cells (ATCC CRL-2266, Manassas, VA, USA) were cultured in high glucose DMEM with sodium pyruvate supplemented with a 10% heat-inactivated FBS and 1% penicillin/streptomycin solution, as described previously [125]. After reaching 80% confluency, the cells were trypsinized (0.05% trypsin/EDTA solution), manually counted in a Burker chamber, and seeded into 96-well plates at a density of 2 × 10^4^ per well in cell culture medium supplemented with retinoic acid (RA, 10 μM). The medium in the plates was exchanged for a fresh one with RA every two days, and cell differentiation was maintained for six days. On the sixth day of culture, the medium was replaced with experimental medium containing DMEM with 1% penicillin/streptomycin solution and 1% FBS. The cells were cultured at 37 °C in an atmosphere containing 95% air and 5% CO_2_ under conditions of saturated humidity.

#### 4.3.3. Primary Neuronal Cell Cultures

Primary cultures of mouse cortical neurons were prepared from 15/16-day embryos originating from pregnant CD1 mice, as described previously [125]. The dissected cortices were digested with trypsin (0.1% for 20–25 min at RT), triturated in the presence of 10% fetal bovine serum and DNase I (150 Kunitz units/mL), and finally centrifuged for 5 min at 100× *g*. The cells were suspended in Neurobasal medium supplemented with B27 and plated at a density of 7.5 × 10^4^ cells per well into poly-ornithine (0.01 mg/mL)-coated 96-well plates. The cultures were then maintained at 37 °C in a humidified atmosphere containing 5% CO_2_ for 7 days before experimentation. This procedure typically yields cultures that contain >90% neurons and <10% supporting cells, as confirmed by immunocytochemistry in our previous study [125].

#### 4.3.4. Cell Treatment

SH-SY5Y cells after differentiation with RA and 7 DIV cortical neurons were treated with cinnarizine (Cin, 10 and 100 μM) alone or in combination with Cin (1–100 μM) and Lac or rotenone (Rot) for 48 h and 24 h, respectively. The used concentrations of cell-damaging factors were Lac 5 μg/mL for SH-SY5Y cells and Rot 10 μM and 1 μM for SH-SY5Y cells and cortical neurons, respectively, which were optimized in our previous studies [125,127].

Rot (10 mM) stock solution was prepared in dimethyl sulfoxide (DMSO). Cin (50 mM) stock solution was prepared in 0.1 N HCl, and pH was adjusted to neutral with NaOH. Lac was diluted in distilled water. All chemicals were added to the culture medium at the indicated concentrations, and solvent was present in cultures at a final concentration of 0.1%.

#### 4.3.5. Measurement of Lactate Dehydrogenase (LDH) Release

To estimate cell death, the lactate dehydrogenase (LDH) level released from damaged cells into the culture media was measured as described previously [125]. The absorbance of the probes was measured at 490 nm using a multi-well plate reader (Multiscan; Thermo Labsystems, Vantaa, Finland). Data were normalized to the LDH level from vehicle-treated cells (100%) and expressed as a percent of the control ± S.E.M. established from two independent experiments with five replicates each.

#### 4.3.6. Measurements of Cell Viability

Cell viability was measured using a tetrazolium salt colorimetric assay with MTT as described previously [125]. The absorbance of probes was measured at 570 nm in a multi-well plate reader (Multiscan; Thermo Labsystems, Finland). The data were normalized to the absorbance of the vehicle-treated cells (100%) and are expressed as a percent of the control ± S.E.M. established from two independent experiments with five replicates each.

### 4.4. Calculation and Statistics

The total rate of DA catabolism was calculated from the ratio of the common DA metabolite HVA to DA concentration and was expressed as an index of the catabolism rate, HVA/DA × 100. To assess the participation of the monoamine oxidase (MAO)-dependent oxidative pathway of DA catabolism, the ratio of DOPAC to DA was calculated and presented as an index of DOPAC/DA × 100. The catechol-O-methyltransferase (COMT)-dependent methylation pathway was assessed likewise, and the index 3-MT/DA × 100 was calculated. The total rate of 5-HT catabolism was calculated from the concentration ratio of its metabolite 5-HIAA to 5-HT and was expressed as the catabolism rate index (5-HIAA/5-HT) × 100. The indices were calculated using concentrations from individual tissue samples. Statistical analysis was performed using STATISTICA 10.0 software (Statsoft, Inc., Tulsa, OK, USA). The significance of differences in the concentrations of DA, 5-HT, and their metabolites between the examined groups and in vitro data were analyzed with a one-way ANOVA followed by a Newman–Keuls post hoc test.

The significance of differences in the concentrations of TH protein levels in the SN of the examined groups and histological data on the number of TH-ir or CV-staining neurons was analyzed with a two-way ANOVA followed by a Newman–Keuls test. *p*-values of ≤0.05 were considered statistically significant. Figures illustrating all presented data were created using GraphPad Prism 9.1.2. software (GraphPad Software, San Diego, CA, USA).

Biochemical data from the in vitro part of the study, after normalization as a percentage ± S.E.M., were analyzed using STATISTICA 10.0 software (Statsoft, Inc., Tulsa, OK, USA). The one-way ANOVA and post hoc Duncan test for multiple comparisons were used to show statistical significance with an assumed *p* < 0.05.

## Data Availability

Data supporting the reported results are available on request from the corresponding author.

## Supplementary Materials

The supporting information can be downloaded at: https://www.mdpi.com/article/10.3390/ijms26188833/s1.

## Abbreviations

The following abbreviations are used in this manuscript:

5-HIAA	5-hydroxyindoleacetic acid
5-HT	serotonin
6-OHDA	6-hydroxydopamine
Calb	calbindin
COMT	catechol-O-methyltransferase
CV	cresyl violet
DA	dopamine
DAT	dopamine transporter
DOPAC	3,4-dihydroxyphenylacetic acid
HPLC	high-performance liquid chromatography
HVA	homovanillic acid
LDH	lactate dehydrogenase
L-VGCC	L-type voltage-gated Ca^2+^ channels
MAO	monoamine oxidase
MPTP	1-methyl-4-phenyl-1,2,3,6-tetrahydropyridine
MT	3-methoxytyramine
MTT	3-[4,5-dimethylthylthiazol-2-yl]-2,5-diphenyltetrazolium bromide
PD	Parkinson’s disease
SN	substantia nigra
SNc	substantia nigra pars compacta
TH	tyrosine hydroxylase
TH-ir neurons	tyrosine hydroxylase immunoreactive neurons
T-VGCC	T-type voltage-gated Ca^2+^ channels
UPS	ubiquitin-proteasome system
VGCC	voltage-gated Ca^2+^ channels
