# Supplementary material for: Cinnarizine, a Calcium Channel Blocker, Partially Prevents the Striatal Dopamine Decline and Loss of Nigral Dopamine Neurons in the Lactacystin-Induced Rat Model of Parkinson’s Disease
Source: Int J Mol Sci. 2025 Sep 10;26(18):8833. doi: 10.3390/ijms26188833 (PMC12469328; doi:10.3390/ijms26188833)
Supplement: Supplementary file 1 [file ijms-26-08833-s001.zip › ijms-3822961-supplementary.pdf]

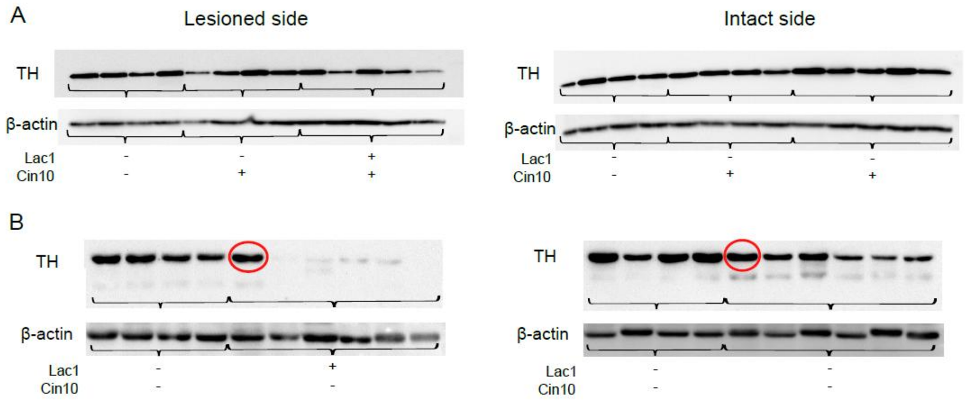


**Figure S1.** Western Blots of TH protein in the lesioned and intact SN of rats originating from the tested groups.

Due to limited space on the gel, Western blot analyses of TH protein levels in the studied groups were performed in two separate experiments. In the first experiment, samples from three groups of rats were analyzed separately for the lesioned and intact sides **(A)**. The second experiment analyzed samples from two additional groups similarly **(B)**. Bands corresponding to individual groups are marked with brackets. In the Lac1-treated group, the bands marked with a red circle (outliers) were excluded from analysis due to an insufficient lesion, as determined by DA content in the lesioned striatum. TH protein levels in all groups were normalized by calculating the TH/β-actin ratio for each sample. Since the normalized TH levels in the control groups (labeled as Lac1- and Cin10- in both **A**,**B**) did not differ significantly, the mean normalized TH protein levels, expressed as a percentage of the control TH/β-actin ratio, are presented together in Figure 4.
